# Detection and characterization of 3D-signature phosphorylation site motifs and their contribution towards improved phosphorylation site prediction in proteins

**DOI:** 10.1186/1471-2105-10-117

**Published:** 2009-04-21

**Authors:** Pawel Durek, Christian Schudoma, Wolfram Weckwerth, Joachim Selbig, Dirk Walther

**Affiliations:** 1 Max-Planck Institute of Molecular Plant Physiology, 14476 Potsdam, Germany; 2 Institute of Biochemistry and Biology, University of Potsdam, 14476 Potsdam, Germany; 3 Molecular Systems Biology, University of Vienna, Althanstraße 14, 1090, Vienna, Austria

## Abstract

**Background:**

Phosphorylation of proteins plays a crucial role in the regulation and activation of metabolic and signaling pathways and constitutes an important target for pharmaceutical intervention. Central to the phosphorylation process is the recognition of specific target sites by protein kinases followed by the covalent attachment of phosphate groups to the amino acids serine, threonine, or tyrosine. The experimental identification as well as computational prediction of phosphorylation sites (P-sites) has proved to be a challenging problem. Computational methods have focused primarily on extracting predictive features from the local, one-dimensional sequence information surrounding phosphorylation sites.

**Results:**

We characterized the spatial context of phosphorylation sites and assessed its usability for improved phosphorylation site predictions. We identified 750 non-redundant, experimentally verified sites with three-dimensional (3D) structural information available in the protein data bank (PDB) and grouped them according to their respective kinase family. We studied the spatial distribution of amino acids around phosphorserines, phosphothreonines, and phosphotyrosines to extract signature 3D-profiles. Characteristic spatial distributions of amino acid residue types around phosphorylation sites were indeed discernable, especially when kinase-family-specific target sites were analyzed. To test the added value of using spatial information for the computational prediction of phosphorylation sites, Support Vector Machines were applied using both sequence as well as structural information. When compared to sequence-only based prediction methods, a small but consistent performance improvement was obtained when the prediction was informed by 3D-context information.

**Conclusion:**

While local one-dimensional amino acid sequence information was observed to harbor most of the discriminatory power, spatial context information was identified as relevant for the recognition of kinases and their cognate target sites and can be used for an improved prediction of phosphorylation sites. A web-based service (Phos3D) implementing the developed structure-based P-site prediction method has been made available at .

## Background

Protein phosphorylation is a ubiquitously occurring post-translational modification influencing many molecular processes in all complex cells. The recognition of phosphorylation sites by specific kinases and the subsequent phosphorylation generally leads to an alteration of the structure, function, or protein binding properties of the target protein, which has evolved as a mechanism to respond to environmental changes via phosphorylation-triggered complex signaling networks and cascades and is playing a crucial role in the regulation of enzymes or transporters in metabolic processes [1-4].

The study of phosphorylation events has been a central research topic in molecular biology for many years. Given the high number of candidate phosphorylation sites, efforts to experimentally identify and verify them all remain challenging. These difficulties motivated the development of computational methods to predict potential phosphorylation sites *in silico*. Most established computational prediction methods rely solely on the local sequence surrounding the target amino acid residue. The developed prediction methods range from simple amino acid sequence pattern recognition methods to Markov Models, Neuronal Networks, and advanced machine learning methods such as Support Vector Machines [5-10]. Many of them have been made publicly available and yield results with reasonable sensitivity and specificity, but they generally suffer from either over- or undercalling candidate sites as optimal parameters found for one particular protein target class cannot be generalized to all phosphorylation motifs [9,11]. Recognizing that the information content increases significantly when the respective kinase families associated with their targets are considered separately, approaches to predict phosphorylation sites in a kinase-family specific manner based on family-specific local sequence motifs have also been presented [5-8].

The acceptable performance of local-sequence-only methods, together with reports that phosphorylation sites appear to be preferentially located in unstructured regions of proteins suggesting a limited relevance of any structurally well-defined binding epitopes for the specific recognition of kinases and their substrate proteins [10], appear to justify focusing exclusively on local sequence patterns rather than three-dimensional (3D)-structural context information. However, the significantly increased number of experimentally determined phosphorylation sites by proteomics technologies with simultaneously available 3D structures of the associated proteins in recent years and published analyses suggesting that target sites may very well assume defined structural conformations and, furthermore, that phosphorylation sites may be surrounded by specific 3D-structural environments [12,13] motivated us to re-investigate the role of 3D-structural information for the specific recognition of kinases and their substrate proteins.

In a recently published systematic comparative and structural analysis of protein phosphorylation, Jiménez and co-workers [12] reported that serine and threonine phosphorylation sites exhibit only a marginal tendency to occur preferentially in structurally more flexible loops with approximately 35% actually being located in α-helices or β-strands, which can be assumed as relatively rigid secondary structural elements. And for tyrosine sites, no tendency to occur more frequently in loops was detectable at all. Furthermore, they reported that a substantial number of phosphorylation sites (15%) are actually buried inside the protein and not exposed to the solvent. An increased significance of 3D-structural context for these locations is evident. Plewczynski and co-workers reported that as many as 60% of phosphorylation sites for the kinase families protein kinase A and C (PKA, PKC) are located in α-helical regions [8]. Thus, a significant number of phosphorylation sites are actually located in structurally defined regions in which defined structural surface features and motifs may turn out to be relevant.

From studying sequence motifs associated with the protein kinase A and G (PKA, PKG kinase families), the consensus target sequence was determined as xRRxSx [14,15]. However, of 273 target motifs for PKA in the Phospho.ELM database [11], 5.5% do not contain any arginine, and 1.5% neither arginine nor lysine in the sequential neighborhood of six residues in both directions relative to the central serine. Of 32 targets for PKG kinases, 9.3% of target sites do not contain any arginine, and in 6% of the targets, both arginine and lysine is absent. This observation implies that some recognition features may perhaps be localized outside of the local sequence, such that the positive-charge bearing amino acids defining the required electrostatic potential surface for binding may be contributed from sequentially distant, but spatially close rather than sequence-local sites.

In the light of these observations, it appears plausible that, although the local amino acid sequence may contain a significant portion of the information contents with regard to phosphorylation, the actual local three-dimensional environment may contribute appreciably to the specificity of the kinase – target protein molecular recognition event.

Although there have been several approaches to use structural information for improved prediction of phosphorylation, they generally resulted in only modest success rates [13,16]. These unsatisfactory results can possibly be explained by an insufficient number of annotated, experimentally determined structures as well as by focusing on general structural properties such as secondary structure, rather than trying to define 3D-motifs based on spatial amino acid distributions.

Fan and Zhang characterized phosphorylation sites in their spatial, protein-structural context using a simplified "Altman" shell model with a radius of 16 Å and found only minor differences of the amino acid composition around phosphorylation sites compared to average protein composition [13,17]. However, by analyzing phosphorylation sites across all kinase families, any motif that may be specific for particular kinase classes may have been masked. The identification of kinase-family-specific sequence motifs supports this view. These amino acid preferences may also be detectable using a protein structural approach which considers spatial proximity rather than sequence proximity alone.

Plewczynski and co-workers applied molecular modeling to characterize the local structural context of phosphorylation sites [8]. In their approach, protein sequences were compared to a library of short sequence and structure motifs via a sequence matching algorithm, adapted for local 3D-structure prediction. They achieved significantly improved prediction accuracy of phosphorylation events by means of similarity scores to a library of PKA and PKC targets and conclude that "sequence information ought to be supplemented with additional structural context information... for more successful predictions of phosphorylation sites in proteins."

The use of structural information for improved phosphorylation site prediction has also been explored by Blom and co-workers, the authors of the popular sequence-only-based NetPhos predication server [16]. In this approach, probabilities of contacts between Cα atoms of residues within spatial neighborhoods of phosphorylation sites and non-phosphorylation sites were calculated, so called contact or distance maps. In a second step, the probabilities of contacts of residues from sequences are then calculated according to those maps and used for prediction purposes. This led to markedly improved sensitivity of the prediction of phosphorylated tyrosine sites which the authors interpreted as an indication of the relevance of tertiary structural information not reflected in the sequence alone. However, this approach also led to an increase of false positive sites and, as a consequence, to overall worse prediction results.

The goal of this work was to characterize phosphorylation sites by spatial amino acid propensity distributions to generate spatial signature motifs and the subsequent assessment of this information to improve the prediction of phosphorylation sites in proteins.

As previous studies have shown that "one-fits-all" approaches; i.e., parameterization of the prediction method irrespective of kinase-family, have led to only modest success rates, we investigate here whether considering kinase-family specific 3D-motifs may reveal greater information contents and, thereby, yield improved prediction results. Our method is based on Support Vector Machines (SVM) [18,19]. SVMs have been used in a wide range of problems in the area of molecular biology including analyses of microarray data [20-22], string matching [23,24], drug design [25], protein fold recognition [26] and prediction of phosphorylation sites using sequence information [7,8,16].

We observed that 3D-motifs are indeed detectable, especially when studying kinase families individually and obtained improved prediction results by including 3D information in the prediction. We also implemented a sequence-only approach that implicitly captures 3D structural preferences associated with each of the different amino acid types by using 530 amino acid features which include also the generally accepted phosphorylation site features such as hydrophobicity, solvent accessibility as well as secondary and tertiary structure preferences, polarity, volume and solvent accessibility, structural disorder indices and others. This predictor has recently been developed by our group as part of a database of plant-specific phosphorylation sites. The predictor was shown to accurately identify plant phosphorylation sites and to outperform commonly available predictors [27].

## Results

**Figure 1:**
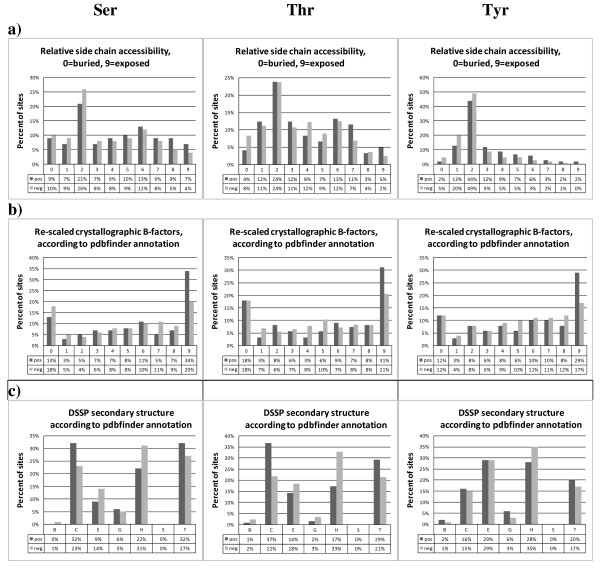
**Comparison of general structural properties associated with phosphorylated (pos.) vs. non-phosphorylated (neg.) residues**. Serine: left column, threonine: middle column, tyrosine: right column. Annotations were taken from PDBFINDER [47]. (a) Side chain accessibility to solvent relative to the large possible accessibility for serine. (b) Re-scaled crystallographic B-factors describe the attenuation of x-ray scattering caused by thermal motion or quenched disorder and is applicable measure for local structural rigidity. B-Factors from PDB-structures in the range of [10,40] are mapped to the range [09] by PDBFINDER; 0 signifying rigid structures, 9 – indicating unresolved, rather flexible structural regions. (c) DSSP secondary structure association. B = residue in isolated beta-bridge; C = Loop, irregular stretches; E = extended strand, participates in beta ladder; G = 3-helix (3/10 helix); H = alpha helix; S = bend; T = hydrogen bonded turn.

To characterize the general structural properties of phosphorylation sites (phos-sites) and to compare them to unphosphorylated sites (non-phos sites), we first analyzed secondary structural assignments, relative side chain solvent accessibility, and the crystallographic B-factor as a measure of local structural rigidity. A statistically significant tendency for serine as well as tyrosine phosphorylation sites to be more exposed to the solvent was detected. Threonine sites were also more exposed, albeit statistical significance could not be established (Figure 1, Table 1). While these observations follow the intuitive expectation that phophate-group attachment sites should be more exposed, the magnitude of the difference appears surprisingly low (Figure 1). However, one has to bear in mind that serine, threonine, and tyrosine – polar amino acids themselves – have an innate tendency to be exposed to water. The distributions of crystallographic B-factors of phos-sites in comparison to non-phos sites were also observed to differ (Figure 1). Phos-sites were more often found associated with the largest B-Factor (Bin 9), i.e. regions of greater structural flexibility, albeit significant *p*-values of differences were only observed for serine sites (Table 1). Phosphorylated serines and threonines are more frequently found in random coil regions and less in α-helical or β-strand regions than their unphosphorylated counter-parts (Figure 1). For tyrosine, no such preferences for secondary structural type were detectable, except for a marginally increased frequency of phosphorylated sites to occur more often in turns.

**Table 1:** Structural features of phosphorylation sites

	**Property**	**mean-Values** **Positive set**	**mean-Values** **Negative set**	**p-Values** **t-Test**	**p-Values** **Mann-Whitney**
**Serine-sites**					
	Accessibility	4.25	3.70	1.32 E-03	1.93 E-03
	B-Factor	5.65	4.93	7.40 E-04	2.79 E-04

**Threonine-sites**					
	Accessibility	3.92	3.57	1.49 E-1	2.07 E-01
	B-Factor	5.30	4.76	1.11 E-1	8.26 E-02

**Tyrosine-sites**					
	Accessibility	2.98	2.36	7.33 E-07	2.52E-06
	B-Factor	5.56	5.15	6.96 E-02	2.68E-02

Statistics for significance of the observed differences of solvent accessibility and crystallographic B-factor of phosphorylated (pos) vs. non-phosphorylated (neg) for serine, threonine and tyrosine sites.

### Characterization of the spatial environment of phosphorylation sites

We determined the propensities for the different amino acid residue types to occur in the spatial vicinity of the phosphorylated serines, threonines, and tyrosines, both for the sequential neighborhood as well as for the spatial-environment. By separating the two, our goal was to identify possible 3D-signature motifs. In a third analysis, both contributions were combined to assess the relative contribution of the sequence and structural environment. As explained in the Method section, across all phosphorylation sites, we calculated the propensity values as log-odds ratios of the relative occurrences of amino acid types within distances from 2 to 10 Å from central phosphorylated amino acid residue and display the results in radial-radial cumulative propensity plots (RCP-plots) in which red-colored segments signify statistically significant enrichment relative to a reference set, and blue-colorings depletion.

**Figure 2:**
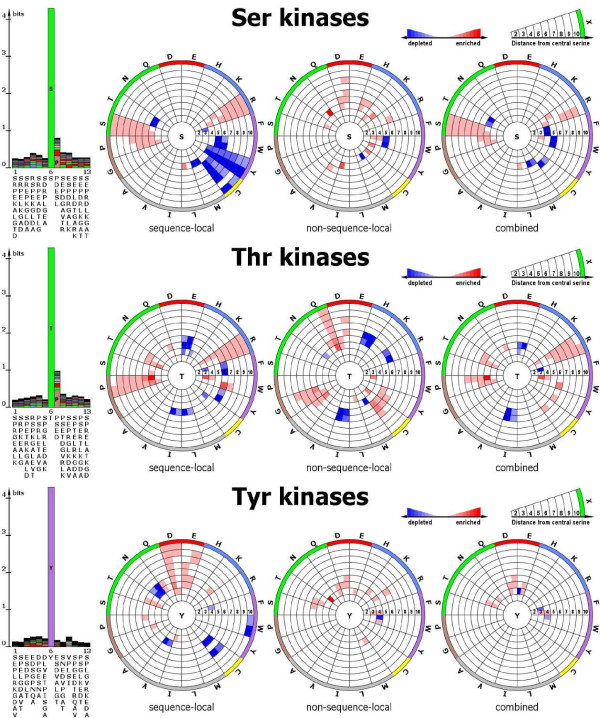
**Sequence logos and radial cumulative propensity plots (RCP-plots)**. Sequence logos and radial cumulative propensity plots (RCP-plots) illustrating enrichment as well as depletion of particular amino acid types in the local sequence (sequence logo), sequence-local spatial environment including the 6 flanking amino acid residues on either side of the central serine/threonine/tyrosine, (left RCP-plot), spatially-local, but non-sequence local; i.e., excluding residues in the flanking sequence (middle plot), and combined information (right-most RCP-plot). For every amino acid type, the two different sub-sectors correspond to the statistics obtained by using the closest detected atom and the interaction center, respectively, and in clockwise order.

When all serine, threonine, and tyrosine phosphorylation sites irrespective of their association with a particular kinase family were analyzed, both the sequence logos and the spatial profile of phosphorylated serines showed only very little information contents (Figure 2). Only small differences relative to the reference set of un-phosphorylated sites were detectable as reflected by the only few colored segments in the RCP-plots indicating enrichment or depletion. For all three target amino acid types, most information appears to be contained in the local sequence and not in the spatial environment. By considering amino acids irrespective of their sequential proximity ("combined" graph), essentially no significant differences to the reference set of un-phosphorylated sites were found. This agrees well with results reported by Fan and Zhang who characterized structural microenvironments of phosphorylation sites within 16 Å from the central residue only and observed no evidence for significant amino acid propensities to fall within radial distance of 16 Å [13]. Interestingly, in the local sequence neighborhood, tyrosine residues – an amino acid that itself is target of phosphorylation events – appear to be depleted relative to the reference dataset in serine- and threonine-targeted phosphorylation sites. However, this depletion appears to be compensated by tyrosine residues found in the spatial environment such that overall ("combined" graph), no significant depletion of tyrosine residues in the environment of serine- and threonine phosphorylation sites was detectable.

### Kinase-family specific phosphorylation motifs

**Figure 3:**
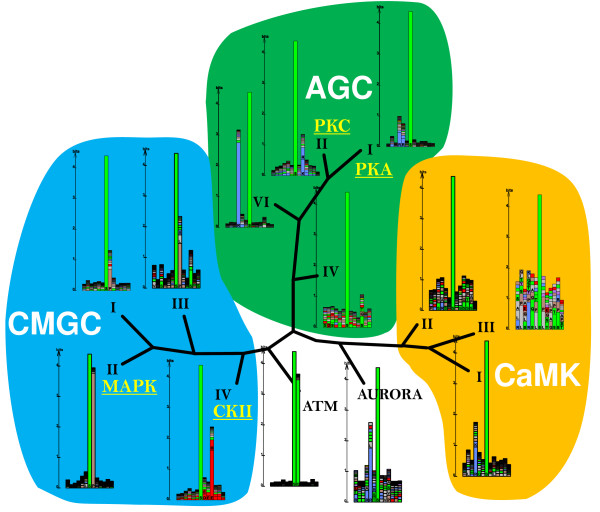
**Phylogenetic tree of serine-kinase groups**. Phylogenetic tree of serine-kinase groups whose targets can be found in the protein structure database (PDB) according to the original Hanks and Hunter classification scheme [45] and associated sequence logos [28]. Kinases with high similarity tend to share similar targets. The major classes of kinase targets are characterized by a proline and glutamate next to the central serine, CMGC group I, II, II and respectively ATM, a group with preferentially negatively charged amino acid residues, CMGC IV and AGC IV, and a large group of targets with an arginine and lysine at the second or third position relative to the central serine, CaMK-Group and AGC-Group except the AGC IV sub family. For kinase families PKA, PKC, as well as CKII and MAPK most targets with resolved structure were available and were used for kinase family-specific predictors in this study.

For the set of serine protein kinase sequences whose target proteins were found by screening the protein structure databank (PDB); i.e., the structure of the target proteins is known, we constructed a phylogenetic tree and computed the corresponding sequence logos of the targets associated with kinase group (Figure 3) [28] to obtain an overview and reference framework of the evolutionary relationships of the kinase sequences and their respective targets. For tyrosine and threonine kinases, respectively, such analysis was not possible (with the exception of the PTK group of tyrosine specific kinases) because of lack of annotated kinase-target pairs with known structure. (Note: A comprehensive phylogenetic analyses of kinases can be found in [29,30]. Here, we focused on kinase-target pairs with determined protein structures of the target protein.) In agreement with results from previous studies, the sequence logos of serine kinase targets associated with the main serine-kinase families can be clustered into several groups [9,16,31,32]. Evolutionarily close kinase-groups tend to also share common features in their respective targets. The major groups of targets are characterized by proline residues next to the central serine (CMGC kinase group except CK II) or a glutamate (ATM), a second group with negatively charged sequences (CMGC IV: CK II). The AGC kinase-group as well as the CaMK kinase-group comprise kinases with positively charged targets.

These enrichments are well captured by the sequence logos and are also reflected in the RCP-plots for the spatial environment considering sequence-local residues. In addition to the detected enrichments (red-colored segments), the RCP-plots also highlight significant depletions of amino acid types (blue segments, Figure 2) that are not immediately apparent from the sequence logo plots alone.

In the following section, we investigate the targets associated with the main kinase families in greater detail. In particular, we are interested to uncover potential 3D-signature motifs beyond the established sequence motifs that can be revealed when investigating individual kinase families rather than across all sites. Such motifs would become evident as colored segments found in the "non-sequence-local" graph, but not found in the "sequence-local" graph. We will refer to those motifs as 3D-signature motifs. Naturally, we limited our analyses to kinase families with sufficient numbers of representatives with the smallest family being the MAPK family with 12 members.

### Serine Sites

#### The AGC group

**Figure 4:**
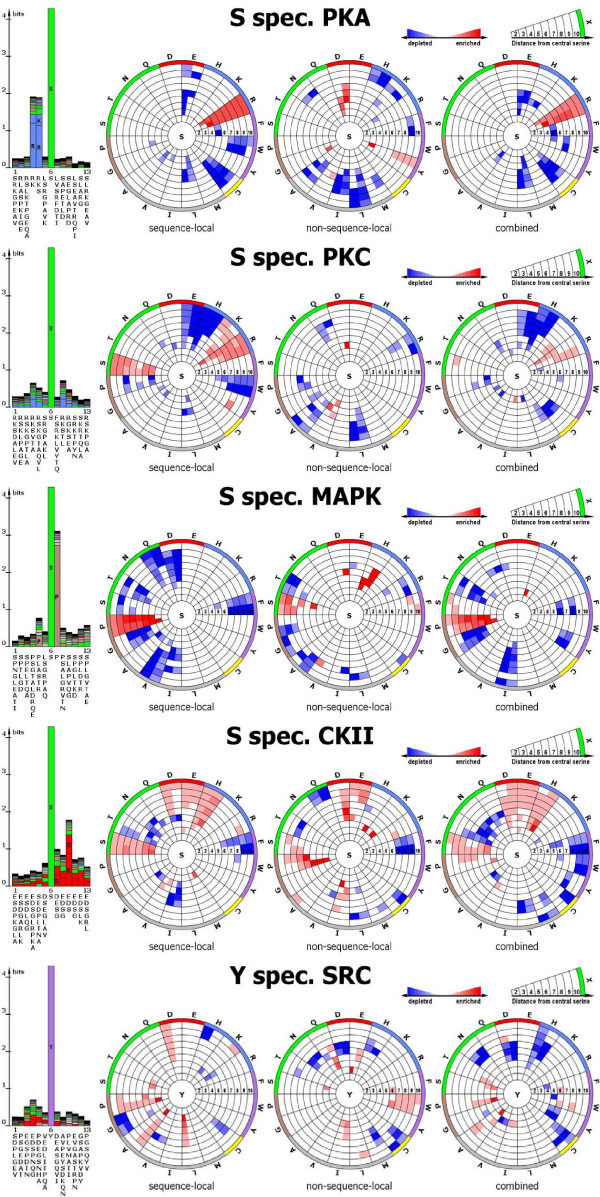
**Sequence logos and radial cumulative propensity plots (RCP-plots) of kinase-specific sequence motifs**. Sequence logos and radial cumulative propensity plots (RCP-plots) of kinase specific sequence motifs, illustrating enrichment as well as depletion of particular amino acid types in the local sequence (sequence logo), sequence-local spatial environment including the 6 flanking amino acid residues on either side of the central serine/threonine/tyrosine, (left RCP-plot), spatially-local, but non-sequence local; i.e., excluding residues in the flanking sequence (middle plot), and combined information (right RCP-plot). For every amino acid type, the two different sub-sectors correspond to the statistics obtained by using the closest detected atom and the interaction center, respectively, and in clockwise order.

The AGC family consists of kinases recognizing serine targets with an arginine or lysine residue at a distance of 2–3 residues relative to the central serine within the local protein sequence and includes the PKA and PKC as well as GRK, BARK, MARK, PKB, PKG, and RSK kinase families which are not included in the study of spatial motifs presented here for paucity of corresponding data. Furthermore, the local sequence-based spatial profile is characterized by lower than expected occurrences of tryptophan and glutamate. Interestingly, the elevated occurrences of the positively charged amino acids arginine and lysine – the hallmark for the AGC kinase group – appears confined to the sequence-local neighborhood. An enrichment of arginine or lysine in the spatial context of PKA was not detectable. In the structural neighborhood ("non-sequence-local" graphs), the counts for both amino acids are not increased relative to the reference distribution. The PKC motifs exhibit an additional enrichment of serine in the sequence-local neighborhood, accompanied by a pronounced depletion of the amino acid residues histidine, glutamate, and tryptophan. The PKA motifs were observed to be depleted of the amino acid cysteine. For both families, PKA and PKC, a depletion of the hydrophobic amino acids alanine and leucine in the non-sequence-local neighborhood and an additional depletion of isoleucine in PKA motifs was detected (Figure 4).

#### The CMGC group

Proline residues flanking the phosphorylated serines are the hallmark sequence feature of targets associated with CMGC kinase group which includes the CDK, CKII kinase families (Figure 3) as well as MAPK and CDC. The CKII and MAPK were included in the spatial study as the number of structurally annotated targets was sufficient. The CKII family from the CMGC IV group, even though grouped into the CMGC group, does not follow the Pro-next-to-Ser rule. Its location in the serine-kinase phylogenetic tree is near the branching point between the CMGC branch and ATM family (Figure 3).

In the sequence-local environment of the MAPK, no enrichments of amino acids besides proline were detectable. Instead, depletions of eight amino acid types, glutamine, asparagine, phenylalanine, isoleucine, valine as well as glycine, serine, and threonine were detected. In the non-sequence-local environment of target serines, serine, and histidine residues were observed to be overrepresented.

The active sites of CKII kinases are characterized by positively charged surfaces [33]. This positive charge density is mirrored by negatively charged aspartate and glutamate in the sequence-local and non-sequence-local spatial neighborhood. Furthermore, the RCP-plots reveal enrichments of serine and histidine the sequence-local and proline in the non-sequence-local RCP pattern. A depletion of phenylalanine is observed at distances of 7 Å and greater for both patterns, while a depletion of threonine, asparagine and isoleucine is only detectable in the sequence-local spatial context.

### Tyrosine sites

#### The PTK group

The PTK group comprises tyrosine phosphorylating kinases and as such were not included in the introduced phylogenetic tree of serine targeting kinases. The sequence-local spatial context of SRC-kinase family (PTK I) – for which sufficient data for analysis was available – is enriched in aspartate, proline, leucine, alanine, and tryptophan in the non-sequence-local spatial context. Depletions of several amino acids were also detectable, most consistently cysteine (Figure 4).

In summary, all kinase-family specific RCP-plots reveal specific spatial profiles and more information contents than was detectable when sites were investigated across all kinase families (Figure 4). The profiles comprise signatures of sequential motifs and discern spatial preferences which cannot be identified by inspecting the local sequence alone. All profiles show significant patterns of enrichments as well as depletions of particular amino acid residue types within the spatial neighborhood of the phosphorylated target amino acid.

### Computational prediction of phosphorylation events using 3D-information

We now turn to investigating whether incorporating 3D structural information can be used to improve the sensitivity and specificity of phosphorylation site predictions in proteins.

### Comparative analysis of prediction performance, Kinase-family-specific predictions

For the general, kinase-family unspecific prediction of phosphorylated serine, threonine, and tyrosine sites, the SVM-predictors based on local sequence information alone that have been developed as part of this study were observed to perform at comparable or even slightly better performance levels compared to NetPhos and DisPhos, and consistently better compared to KinasePhos as judged by the area under the receiver operating characteristic (AUC) from 10-fold cross-validation test (Table 2).

**Table 2:** Prediction performance as measured by the AUC

**Kinase family**	**Kinase group**	**N**	**Sequence-only**	**Spatial-information enriched**	**NetPhos 3.1b**	**DisPhos 1.3**	KinasePhos 2.0*
Ser kinases	**/**	363	0.74 ± 0.02	**0.79 ± 0.02**	0.69 ± 0.02	0.73 ± 0.05	0.63 ± 0.05
PKA	**AGC I**	34	0.91 ± 0.04	**0.94 ± 0.04**	0.91 ± 0.03		
PKC	**AGC II**	31	0.83 ± 0.05	**0.87 ± 0.04**	0.78 ± 0.05		
MAPK	**CMGC II**	12	0.89 ± 0.07	**0.91 ± 0.06**	0.78 ± 0.09		
CKII	**CMGC IV**	19	0.73 ± 0.07	**0.78 ± 0.07**	0.76 ± 0.07		

Thr kinases	**/**	134	0.72 ± 0.03	**0.74 ± 0.03**	0.66 ± 0.03	0.72 ± 0.06	0.66 ± 0.05

Tyr kinases	/	**253**	0.69 ± 0.02	**0.71 ± 0.02**	0.65 ± 0.02	0.56 ± 0.06	0.54 ± 0.05
SRC	**PTK I**	24	0.72 ± 0.07	**0.79 ± 0.06**	0.62 ± 0.07		

							

unspecific predictor		750	0.71 ± 0.01	**0.75 ± 0.01**	0.67 ± 0.01	0.68 ± 0.03	0.63 ± 0.03

Results from the cross-validation of the various prediction approaches. The sequence-only and Spatial-information enriched methods were developed as part of this study and compared to NetPhos 3.1b that includes the kinase-specific predictor NetPhos/K, DisPhos1.3 and KinasePhos2.0. As KinasePhos reports only decision values of positively predicted sites, the evaluation of kinase specific prediction was not possible due to missing score values for sites not predicted to be phosphorylated. However, the kinase-specific predictions were feasible as KinasePhos essentially reports all submitted sites as being phosphorylated by at least one kinase. For the evaluation of the predictor, the highest reported decision value was used for each site. Best performing methods are printed in bold-face.

Similarly, for the kinase-family specific predictions, the AUC-based performance of NetPhos and our SVM-based method was comparable or even in favor of our SVM (Table 2) giving us an appropriate best possible sequence-information-alone baseline to assess the effect of adding 3D-structural information on the prediction accuracy when added to the SVM.

While the magnitude of performance gain when including 3D-profile information was relatively small compared to the estimated standard error, for all target sites and across all kinase-families and target residue types, a consistent increase in performance was obtained suggesting that including three-dimensional structural information does indeed improve the sensitivity and specificity of phosphorylation site prediction.

Similar conclusions can be drawn from comparing prediction accuracies as well as sensitivities and specificities associated with the predictions rather than AUCs (Table 3) alone. Unlike in the case of AUC, where it was impossible to compute AUC values for the KinasePhos 2.0 prediction program because of non-returned score values, here it was possible to obtain relevant values also for the KinasePhos 2.0 prediction program. Again, adding 3D-information to using only sequence information resulted in modest (up to 5 percentage points), yet consistently improved predictions for all three target amino acid types as well as kinase-family specific targets such that best prediction results were always obtained by using our 3D-information enriched SVM-based prediction method with the exception of the kinase families PKC and MAPK for which the performance was virtually identical compared to our sequence-only SVM, but still better than the other prediction programs included in this study. The most significant gain was obtained for serines sites followed by tyrosine and threonine sites.

**Table 3:** Prediction Performance as measured by accuracy, sensitivity (sn), and specificity (sp)

**Kinase family**	**Kinase group**	**N**	**Sequence-only**	**Spatial- information enriched**	**NetPhos 3.1b**	**Disphos 1.3**	**KinasePhos 2.0**
Ser kinases	/	363	0.69 ± 0.01 sn:0.76 ± 0.02 sp:0.62 ± 0.03	**0.73 ± 0.01** sn:0.73 ± 0.01 sp:0.73 ± 0.02	0.64 ± 0.01 sn:0.70 ± 0.00 sp:0.58 ± 0.01	0.68 ± 0.01 sn:0.47 ± 0.00 sp:0.88 ± 0.03	0.50 ± 0.00 sn:1.00 ± 0.00 sp:0.50 ± 0.00

PKA	**AGC I**	34	0.83 ± 0.03 sn:0.93 ± 0.05 sp:0.83 ± 0.07	**0.88 ± 0.02** sn:0.86 ± 0.05 sp:0.80 ± 0.07	0.82 ± 0.02 sn:0.82 ± 0.00 sp:0.81 ± 0.05		0.71 ± 0.03 sn:0.65 ± 0.00 sp:0.75 ± 0.04

PKC	**AGC II**	31	**0.82 ± 0.02** sn:0.76 ± 0.03 sp:0.87 ± 0.03	**0.82 ± 0.02** sn:0.80 ± 0.03 sp:0.81 ± 0.04	0.72 ± 0.02 sn:0.58 ± 0.00 sp:0.86 ± 0.04		0.64 ± 0.03 sn:0.61 ± 0.00 sp:0.66 ± 0.04

MAPK	**CMGC II**	12	**0.89 ± 0.04** sn:1.00 ± 0.00 sp:0.79 ± 0.08	**0.89 ± 0.04** sn:0.88 ± 0.15 sp:0.79 ± 0.08	0.69 ± 0.02 sn:0.42 ± 0.00 sp:0.96 ± 0.04		0.61 ± 0.05 sn:0.50 ± 0.00 sp:0.64 ± 0.08

CKII	**CMGC IV**	19	0.70 ± 0.03 sn:0.79 ± 0.18 sp:0.60 ± 0.18	**0.74 ± 0.04** sn:0.88 ± 0.15 sp:0.61 ± 0.14	**0.74 ± 0.02** sn:0.53 ± 0.00 sp:0.94 ± 0.04		0.62 ± 0.03 sn:0.47 ± 0.00 sp:0.67 ± 0.07

Thr kinases	/	134	0.68 ± 0.01 sn:0.55 ± 0.04 sp:0.87 ± 0.04	**0.69 ± 0.01** sn:0.61 ± 0.04 sp:0.80 ± 0.04	0.63 ± 0.01 sn:0.49 ± 0.00 sp:0.77 ± 0.02	0.66 ± 0.03 sn:0.49 ± 0.00 sp:0.83 ± 0.05	0.50 ± 0.00 sn:1.00 ± 0.00 sp:0.50 ± 0.00

Tyr kinases	/	**253**	0.65 ± 0.01 sn:0.67 ± 0.06 sp:0.63 ± 0.06	**0.67 ± 0.01** sn:0.51 ± 0.03 sp:0.81 ± 0.03	0.62 ± 0.01 sn:0.54 ± 0.00 sp:0.71 ± 0.01	0.53 ± 0.02 sn:0.29 ± 0.01 sp:0.77 ± 0.05	0.50 ± 0.00 sn:1.00 ± 0.00 sp:0.50 ± 0.00

SRC	**PTK I**	24	0.70 ± 0.03 sn:0.74 ± 0.15 sp:0.66 ± 0.16	**0.75 ± 0.03** sn:0.77 ± 0.09 sp:0.72 ± 0.10	0.57 ± 0.01 sn:0.17 ± 0.00 sp:0.98 ± 0.10		0.70 ± 0.04 sn:0.83 ± 0.00 sp:0.66 ± 0.04

							

unspecific predictor		750	0.66 ± 0.01 sn: 0.65 ± 0.01 sp:0.69 ± 0.02	**0.69 ± 0.01** sn:0.60 ± 0.03 sp:0.78 ± 0.03	0.63 ± 0.01 sn:0.61 ± 0.01 sp:0.66 ± 0.01	0.62 ± 0.01 sn:0.42 ± 0.01 sp:0.83 ± 0.03	0.50 ± 0.00 sn:1.00 ± 0.00 sp:0.50 ± 0.00

Results from the cross-validation of the various prediction approaches. The sequence-only and Spatial-information enriched methods were developed as part of this study and compared to to NetPhos 3.1b that includes the kinase-specific predictor NetPhos/K, Disphos1.3 and KinasePhos2.0. The size of the negative set was adjusted to the size of the positive sites, ensuring equal sizes of the sets and a comparison to original reports of accuracies of alternative prediction approaches. In the case of the kinase unspecific prediction of KinasePhos2.0, all sites were predicted to be phosphorylated by at least one kinase. Best performing methods are printed in bold-face. Sn denotes sensitivity, while sp denotes the specificity for the stated accuracy.

## Discussion

In this work, we focused on the characterization and prediction of phosphorylation sites. Serine is the most frequent target amino acid residue type for phosphorylation followed by threonine and tyrosine. We pursued two major themes: the analysis phosphorylation in a kinase family specific fashion, and to investigate whether phosphorylation sites are characterized by specific three-dimensional (3D) structural motifs or epitopes constituted by amino acid residues that are not necessarily close in sequence, thereby providing additional information that can help in predicting phosphorylation sites for proteins with known structure or with available structural models. We used the simple radial distance to define structural motifs. Ideally, angular information would be included as well. However, much larger datasets of determined structures would be necessary to derive reliable statistical data for more refined approaches. Even by applying only this simple model, we observed that 3D-structural context information is indeed discernable, even though the most information contents appears to reside primarily in the local sequence, as judged by the sequence-local kinase unspecific RCP-plots and the modest increased performance when adding spatial-information to sequence-only based predictors. The most pronounced improvement of prediction of phosphorylation sites by augmenting sequence-only prediction by spatial information was obtained for targets of serine kinases. However, also for the prediction of threonine and tyrosine targets, a performance gain was obtained when adding 3D information.

As several experimental techniques have been established to detect proteins that specifically bind to phosphorylated sites based on immobilized peptides (pull-down assays and peptide chips [34-36]); i.e., the binding epitope is reconstituted from the sequence-local amino acid residues alone, the results obtained in this study lend further support to such approaches. Based on the findings obtained for our dataset, spatial information is discernable, but may not be absolutely critical to define the binding epitope, although conclusively proving it will require experimental comparisons of binding efficiencies for known interacting partners based on the complete and natively folded as well as local peptide sequence.

It has been reported that phosphorylation is preferentially occurring in unstructured; i.e., flexible regions of proteins [10]. These conclusions resulted from sequence-based predictions of the flexibility of phosphorylated and non-phosphorylated sites and are also supported by the reasonable prediction performance by DisPhos1.3 for serine and threonine. The prediction of phosphorylation sites by DisPhos is based on a prior predication of local flexibility. However, many phosphorylation sites were found in regions of clearly defined secondary structures (Figure 1). We further investigated this by comparing the crystallographic B-factor as well as secondary structural class for phosphorylated and unphosphorylated serine sites (Figure 1). In the latter, loop regions may represent rather unstructured segments, even though it does not mean that this regions are structurally flexible. Flexibility may be better captured by the reported B-factor. We found statistically significant differences of B-factors for phosphorylated compared to non-phosphorylated serine sites detectable, albeit the differences were not that large. Of course, we only included those proteins in our investigation with an available crystallographic structure; including atomic coordinate information for the targeted peptide segment itself. It may be possible that, by only using fully resolved structures that we needed in order to detect possible 3D-motifs, we excluded phosphorylation events in unstructured regions right from the start. Indeed, 86% of removed motifs (Ser: 88%; Thr: 93%; Tyr: 75%) were localized in loops as judged by prediction by DisEmbl 1.5 [37]. Within the training dataset only 66% of phos-sites (Ser: 68%; Thr: 72%; Tyr: 60%) and 53% of non-phos sites (Ser: 55%; Thr: 53%; Tyr: 51%) were predicted in loops (Table 4). Moreover, 3% of phosphorylated residues were found near the protein sequence termini where the structural flexibility naturally increases, which were not considered as potential target sites. Follow-up studies need to be performed to address this question more systematically by mapping sites that were found in peptide-based methods (mass spectroscopy) and to map them to available protein structures and to gather statistics how often phosphorylation sites map to regions that cannot be resolved crystallographically.

**Table 4:** Predicted ratios of sites in loop regions

**Property**	**removed phos sites**	**training set** **phos sites**	**training set** **non-phos**
All	86%	66% (53%)	53% (45%)
Ser	88%	68% (57%)	55% (54%)
Thr	93%	72% (64%)	53% (47%)
Tyr	75%	60% (41%)	51% (34%)

Predicted ratios of sites in loop regions as judged by prediction by DisEmbl 1.5 [37]. Percent of sites in loop regions according to the annotation of secondary structure of B, C, S, and T by DSSP is given in brackets.

Gnad and co-workers, the authors of the PHOSIDA database, evaluated the preferences of secondary structure, accessibility of phosphorylated residues and the conservation rate of phosphorylation sites [38]. The preferences of secondary structure and accessibility were estimated by prediction. Consistent with our results, they found small, yet statistically significant differences between the phos-site and non-phosite for serine, threonine, and tyrosine motifs. Furthermore, they were able to improve the performance of prediction by applying the predicted values to prediction. Although the average predicted accessibility differed from our results, the tendencies were comparable. The computed average accessibilities in the PHOSIDA-approach were 3.7 and 3.4 for phos and non-phos serines, 3.5/3 for threonines and 2.2/1.7 for tyrosines, respectively. In our approach the accessibilities and the secondary structure preferences were determined based on fully resolved protein structures. For PDBFINDER-based accessibility values, we determined 4.28/3.62 for serines, 3.88/3.52 for threonines and 2.96/2.27 for tyrosines, respectively. Furthermore, Gnad et al. predicted 93% of phosphorylated serines and 78% of non-phosphorylated serines to occur in loops and hinge regions. For threonines, the corresponding frequencies were determined as 90% and 70%, and for tyrosine 79% and 48%, respectively. In our approach, we found significant differences of secondary structural preferences between phos and non phos-sites. In addition, the determined frequencies of sites in rigid regions (non-loop regions) were much higher. However, including accessibility and the secondary structure information did not yield any improvements of the prediction, probably because both these properties were implicitly covered by the amino acid properties already.

A major problem in any effort to develop a computational predictor arises from the difficulty to define a reliable true-negative set; i.e., sites that are truly unphosphorylated. As phosphorylation is condition-dependent, experimental screens may well be incomplete as it is impossible to explore all environmental conditions under which phosphorylation events may occur. Even sites that are buried and inaccessible for phosphorylating kinases in one protein state may become exposed upon conformational changes and become phosphorylated [12,39]. Thus, even buried sites cannot be ruled out as candidate phosphorylation sites. Even more so, the numerical value for solvent accessibility may oftentimes suggest that a serine is buried, even though it is actually a surface residue, but occluded by neighboring side chains and not buried deep in the protein's core. The assumption that buried amino acids cannot become phosphorylated and using it as criterion for the construction of a negative set may, in fact, be misleading. The resulting predictors will tend to predict accessibility of target sites rather than the possibility of phosphorylation. An alternative way for defining negative sets is including of all candidate sites (serine, tyrosine, or threonine residues) except experimentally verified phosphorylation sites with the reasoning that such a true-negative set will at least be depleted in true-positive sites. In this study, we followed this approach, realizing that this may represent a source of error.

An estimated two to five percent of eukaryotic genomes codes for kinase genes grouped into different kinase families [29,30] and 30% of all proteins are estimated to be phosphorylated as judged by proteomics screens [15,40]. Mirroring the many different kinases catalyzing the addition of phosphate group to proteins, the high diversity of their cognate phosphorylation target sites is a major obstacle for a reliable prediction of phosphorylation. In addition, experimental evidence suggests that the kinases are to some degree unspecific and are capable of phosphorylating a wide spectrum of substrates [15]. On the other hand, evidence for sequence-encoded specificity on the side of phosphorylation target has also been presented. For example, the prediction accuracy of phosphorylation sites in plant proteins was shown to increase substantially when the computational methods were trained on plant proteins versus methods trained primarily on animal proteins suggesting kingdom specific differences of phosphorylation target sites [27,41].

The high diversity of targets of particular kinases and the number of possible phosphorylated proteins accompanied with the pleiotropicity of kinases appear to contradict a specific regulatory role of phosphorylation. However, the specificity for the actual target site may not be the only source of kinase specificity and sensitivity of the regulatory system. In fact, it was shown that subcellular compartmentation accompanied with recognition of secondary target sites relatively distant to the catalytic domains is crucial for further selectivity and specificity. While 3D motifs near the actual target site for phosphorylation have been at the center of our investigations, for the kinase family CDK, in particular the kinase CDK2 [42], it has been reported that secondary sites, protein surface site distant from the actual phosphorylation site may determine binding specificity of kinases with their target protein. Therefore, the systematic identification and characterization of such secondary recognition sites appears worthwhile [43]. Kinase activation in kinase cascades by post translational modification, formation of protein complexes as well as priming of phosphorylation further enhance the sensitivity of the phosphorylation system [15].

## Conclusion

The reliable prediction of phosphorylation sites and the identification of associated kinase enzymes are important steps that will ultimately lead to a deeper understanding of complex signaling events in cellular systems. Applying a simple radial distance model for the characterization of the 3D-structural context of phosphorylation sites, it is possible to extract kinase specific signature 3D-profiles. While local one-dimensional amino acid sequence information was observed to harbor most of the discriminatory power, spatial context information was identified as relevant for the recognition of kinases and their cognate target sites and can be used for an improved prediction of phosphorylation sites. A web-based service (Phos3D) implementing the developed structure-based P-site prediction method has been made available at .

## Methods

### Creation of phosphorylation site dataset (phos-Set)

The dataset of phosphorylation sites was obtained from the Phospho.ELM database [11]. The amino acid residue annotated as phosphorylated (Ser/Thr/Tyr) was placed in the middle position of the 13-mer peptide with six amino acid residues on either side flanking the central position extracted from the native sequence of the respective protein harboring the site. Incomplete (i.e. truncated) motifs were discarded. The data set comprised 14,630 non-redundant sequence motifs (10,769 serine, 2,095 threonine, and 1,765 tyrosine motifs). To identify associated protein structures and the actual conformations and locations of the motifs within their three-dimensional context, we screened the Protein Data Base (PDB) for protein structures containing the 13-mer peptide sequence associated with phosphorylation sites based on exact sequence matches. We found 1,234 motifs (Ser: 633, Thr: 241 and Tyr: 360), which corresponded to 14,192 exact matches (Ser: 6,757, Thr: 2,757, and Tyr: 4,865 matches) in 6,596 different PDB-protein chains (Ser: 4,337, Thr: 2,086, Tyr: 2,765 chains); i.e., many motifs were found multiple times in different PDB-protein structures. The identified protein structures corresponded to 704 unique phospho-proteins (Ser: 430, Thr: 227, Tyr: 202) as judged by their corresponding SWISS-PROT [44] identifier. The dataset consisted of highly non-redundant protein sequences as evidenced by the low levels of sequence identities (Additional file 1). Considering only structures with complete atomic coordinates of amino acids in their non-phosphorylated state for the phosphorylation motif and choosing the structure with the best crystallographic resolution in case of identical sequence motif hits, we obtained a final set of 750 non-redundant, structurally resolved phosphorylation motifs (Ser: 363, Thr: 134, Tyr: 253 structural motifs). For a subset comprising 307 motifs (Ser: 164, Thr: 59, Tyr: 84 motifs), information of their respective phosphorylating kinases was available, and the associated motifs were classified into respective kinase families. All motifs as well as the associated PDB entries and annotated kinase family annotations are provided in Additional file 2.

### Creation of a non-phosphorylation site datasets (non-phos-Sets)

We removed the phos-Set motifs from the sequences of the respective protein chains with known protein structure. From the remaining sequence fragments, we extracted all non-overlapping Ser/Thr/Tyr site motifs. The resulting sets of sites served as the true-negative set. While our approach cannot guaranty that these extracted sites are truly unphosphorylated, we expect this dataset to be at least depleted in true phosphorylation sites.

When kinase-family-specific phosphorylation events are analyzed, the true-positive counts are heavily outnumbered by true-negative sites posing the risk of dominating influences of the negative set rather than the positive set. In particular, the false-negatives; i.e., sites that we grouped as unphosphorylated that may, however, become phosphorylated under different conditions may then obscure any discernible signal. To alleviate this problem, while at the same time keeping a sufficient number of examples for training purposes, we reduced the negative set for kinase specific predictions by randomly eliminating sites from the non-phos-Set until the negative sets were no more than twice as large as the positive sets.

### Construction of the phylogenetic tree of serine-kinases

Sequence motifs associated with putatively phosphorylated serines are partly annotated with their respective phosphorylating kinase and can be grouped into kinase families and groups according to the classification scheme proposed by Hanks and Hunter augment by the AURORA and ATM kinase group [45]. Considering only kinase groups with known targets, a phylogenetic tree (dendrogram) was built from representative sequences using the CLUSTALW package [46]. For each group, we calculated sequence logos from all respective targets, i.e. also targets which are not represented in the protein database PDB [28]. A more in-depth analysis of the phylogeny of human kinases is provided in [30].

### General structural properties of phosphorylated and unphosphorylated sites

Secondary structural assignments, relative side chain accessibilities, crystallographic B-Factors were obtained from the PDBFINDER II database [47-50].

### Calculation of spatial amino acid propensity profiles, Radial Cumulative Propensity (RCP) plots

Propensity ratios (odds-ratios) defined by the normalized counts of a particular amino acid type around sites in the phos-Set relative to their counts observed around sites in the non-phos-Set representative set within radial distances ranging from 2 to 10 Å from the central Ser/Thr/Tyr were calculated according to Equation 1. The chosen distance range covers both direct contacts as well as through-space interactions such as electrostatic interactions. Beyond 10 Å, we did not find any significant enrichment or depletion signals. We used two different distance measures. Amino acid residues were considered to lie within a given radial cutoff distance if i) the distance between the putatively activated oxygen (β-hydrogen) in case of a central serine and threonine, or γ-carbon in case of tyrosine and any atom of that residue was shorter than the given cutoff distance, or, if ii) the distance between the interaction centers of residues as proposed by Park et al. [51] fell within a given radial distance cutoff. The proposed interaction centers were shown to better represent interactions between amino acid residues associated with secondary structural elements within proteins. Furthermore, both distance measures represent a different degree of resolution of atomic detail. Radial distance-dependent propensity ratios for all 20 amino acid types are illustrated graphically in radial cumulative propensity plots (RCP-plot). These plots reflect the cumulative spatial amino acid residue propensity profile around phosphorylation sites. We differentiate between radial profiles associated with i) sequence-local amino acids, i.e. amino acid residues located within 6 residues from the central serine in the protein sequence, and ii) non-local amino acid residues; i.e., residues that are outside the local sequence environment (> 6 residue positions), and, iii), the general spatial profile irrespective of the amino acid position in the protein sequence. The 20 radial sectors associated with the different amino acid types are divided into two sub-sectors according to the two different distance measures used. The significance of the obtained propensities for increased or decreased occurrences relative to random expectation around phosphorylated sites was assessed by estimating the standard error of the odds-ratios, S_E_, as proposed by Levitt (Equation 1) [52]. Odds-ratios signifying over- or underrepresentation were considered statistically significant if odds-ratio > 2 and (odds-ratio - *S*_*E*_) > 1 with odds-ratios inverted in cases where the propensity ratio was below 1; i.e., observed less than expected by chance.

(1)

Calculation of amino acid propensity ratios for the estimation of average depletion or enrichment given a particular motif set. #*AA*_*k*/*s *_is the count for amino acids, where *k *designates a particular amino acid residue type and *s *is the count summed over all amino acid residue types; *r *is the considered radial distance to the central serine/threonine/tyrosine, *f *is the relative frequency of amino acid in a particular set, and *g *the relative frequency of the amino acid *k *in the reference non-phos set. Associated standard errors *S*_*E *_were estimated according to [52].

### Prediction approach, evaluation of prediction performance

To predict phosphorylation sites from sequence and to evaluate the effect of using structural information on prediction performance, we applied Support Vector Machines (SVM), first using sequence information alone and, subsequently, enriched by the spatial information. We used the "kernlab" R-package developed by Alexandros and co-workers [53] applying the default Radial Basis kernel with automated sigma estimation. We evaluated the Area Under the Receiver Operator Characteristic (ROC)-curve (AUC) from a 10-fold cross-validation to quantify the performance of predictors and to compare the obtained results to prediction results obtained by using NetPhos and DisPhos [9,10,16]. Associated standard errors were computed as in [54].

The 10-fold cross-validation was based on training of the predictor on 9 out of 10 parts of the randomly ordered data set and subsequent classification of the remaining part. The test is repeated for all 10 possible partitions of the dataset. The classification results are then used for measuring the performance of the predictor. The developed classifiers based on Support Vector Machines included general, kinase-family unspecific serine, threonine, and tyrosine predictors; i.e., the parameters were trained across all proteins irrespective of annotated kinase family, as well as predictors specific for the serine-centric PKA, PKB, MAPK, and CKII kinase family as well as tyrosine-centric SRC kinase family, for which at least 10 annotated targets or more were contained in the dataset. The minimal number of targets allowed a 10-fold cross-validation with the lowest actual number being 12 kinase-annotated targets (MAPK). For threonine target sites, the respective kinase-family annotation information yielded only data sets of insufficient size for statistical analyses. The area under the ROC-curve (AUC) from 10-fold cross-validation was compared among different prediction approaches and programs to judge, whether the addition of spatial information can improve the prediction performance. Perfect prediction results would yield an AUC of 1, while guessing the outcome would, on average, yield AUCs of 0.5.

### Feature-vectors (FV) for the implemented Support Vector Machines

The feature-vector (FV) used for the Support Vector Machines consisted of chemical-physical amino acid properties for the sequence-information-only approach and an additional spatial information component for the spatial prediction approach. For the amino acid property components of the FV, we utilized values from the collection of 530 commonly used indices provided by the AAindex database [55] including hydrophobicity, solvent accessibility preferences, secondary and tertiary structure preferences, polarity, volume and solvent accessibility, structural disorder indices and others. The vector consisted of 530 × 12 dimensions for every index and position around the central serine, threonine, or tyrosine, where the components were values from the respective index and 530 dimensions for the average index value of the particular sequence motif. The latter dimensions were introduced to cover the general properties of the motifs, e.g. negative charge or high flexibility. To reduce the dimensionality of the Feature-vectors (FV) as well as to eliminate correlations between components, principle component analysis (PCA) was performed on the *D*_*FV *_× *N *data matrix, where *D*_*FV *_is the number of components of the Feature Vector, and *N *is the number of example peptide sequences in the training set, and the components of the FV were replaced by the resulting principle components with non-zero Eigenvalues explaining the entire variance in the dataset. Note: as there are fewer examples (*N *peptide sequences in the training set) than dimensions, the dimensionality (Eigenvectors with non-zero Eigenvalues) can be at most *N*-1. The PCA was performed independently for the serine, threonine, and tyrosine motifs. The total variance contained all independent datasets was essentially completely covered by 228 principal components. This low number of PCs (compared to 530 properties) results from the high correlation of amino acid indices. Although computed by different approaches, various indices designed to capture different properties also show similar tendencies, e.g. hydrophobicity and polarity. Moreover, apparently different properties of amino acids, like hydrophobicity and solvent accessibility are based on similar attributes of amino acids and, therefore, also correlate. In addition, the preference for a particular secondary structure and structural flexibility correlate to a high degree. In this case, however, the correlation is negative and the respective loadings on the PC have different signs. By evaluation of the loading matrix from the PCAs, we observed that the PCs are mostly influenced by the hydrophobicity and flexibility values, which appear most often in combination. The PCs differed with regard to the particular position of the property, rather than to the loadings of different indexes. For serine sites, PC1 was most influenced by the sum of the hydrophobicity and flexibility values over the entire sequence motif, PC2 by these properties at positions -6/+2, and PC3 at positions +1/+4 and PC5 -3/+3. PC4 included the variance of amino acid propensities to rigid structures at position -1 and the sum of these propensity values. The PCs of the PCA of threonine sites were based on the variance of the sums of the hydrophobicity and accessibility index values for PC1 and sum of the amino acid propensities to rigid secondary structures for PC2. Variances of hydrophobicity and flexibility at position +4 were loaded in PC3, +5 in PC4 and +6 in PC4. For tyrosine sites we found the sum of the hydrophobicity and flexibility indices in PC1, at position +3 in PC2, the sum and position +1 of preferences to rigid secondary structures in PC3, -1/+1 hydrophobicity in PC4 and hydrophobicity, accessibility, and polarity at position -6 in PC5. For a comprehensive analysis, we provide the complete data matrix of variable (amino acid index) loadings as part of the newly developed Phos3D prediction server .

The spatial information component consisted of the normalized distribution ratios according to (Eq. 2). The ratios of amino acid residues within the local sequence, outside the local sequence, and irrespective of the position in the protein sequence were used for distances in a range of 2 to 10 Å between the putatively activated oxygen (β-hydrogen) in case of a central serine and threonine, or γ-carbon in case of tyrosine and the closest atom of all other amino acid residues, or between the interaction centers proposed by Park and coworkers [51].

(2)

Radial distance odds-ratios for particular amino acid residue types as explained in Eq. 1 with an additional rescaling rendering resulting values suitable for use in kernlab package by ensuring that values lie between zero and one and with 0.5 indicating a balanced count (neither over-, nor under-representation) designated as *oddsSVM*.

### Comparison to NetPhos, DisPhos-1.3 and KinasePhos2.0

We compared the AUC from the 10-fold cross-validation results obtained by using NetPhos, NetPhosK, DisPhos, KinasePhos2.0. NetPhos and NetPhosK are both part of the NetPhos-3-1b package. While NetPhos was designed to generally predict serine, threonine, and tyrosine phosphorylation events, NetPhosK includes kinase-specific predictors. DisPhos is based on SVM, utilizing the binary representation of the motif sequence, the relative frequencies of amino acids in that sequence as well as outputs from predictors for structural disorder and secondary structure[10]. Furthermore, the Feature Vectors are supplemented by amino acid properties covering the sequence complexity, net-charge and aromatic content, hydrophobic moment, and hydrophobicity as well as values according to a flexibility and surface exposure scale. Thus, the features used by DisPhos are comparable to features applied in our predictors. While NetPhos and DisPhos are predictors for phosphorylation events, KinasePhos2.0 was developed to identify the respective kinase [56], comprising over 50 kinase-specific predictions. The server is reported to yield highly accurate results also for the general prediction of phosphorylation events and, therefore, a good benchmark for the kinase specific predictors developed here.

For comparison with DisPhos, we submitted 60 randomly selected protein sequences covering at least 50 positive and 100 negative motifs for serine, threonine, and tyrosine sites to the DisPhos 1.3 server [57]. Although 60 protein sequences are only a small subset of the total of 869 protein structures, the sequences cover 14% serine, 37% threonine and 20% tyrosine sites. Sites being reported as predicted by similarity to the training sequences, as assigned by DisPhos were removed to avoid self-recognition. A similar procedure was applied to KinasePhos 2.0, however the KinasePhos2.0 server as well as NetPhos do not provide information of possible self-recognition events. We submitted the above mentioned protein sequences to the KinasePhos 2.0 server [58] setting the specificity value to "default". The comparison proved difficult as only positively predicted (phosphorylated) sites, i.e. sites which were predicted by a decision value above 0.5 were returned. This rendered the computation of the AUC for specific predictors impossible, as not for all submitted sites, a decision value (score) was available. However, as essentially all sites from the training and test set, irrespective of whether they were positive or negative were predicted to be phosphorylated by the server by at least one kinase, assessment of the performance of the prediction by evaluation of the AUC for kinase unspecific prediction was still possible. Out of 1,335 submitted serines, 1,288 (97%) were predicted as being phosphorylated. The corresponding ratios for threonines were 1,098 out of 1,124 (98%), and tyrosines 713 out of 723 (98%). Before evaluating the ROC curve, for each site, the highest reported decision values were determined. For a meaningful comparison, the size of the results from DisPhos and NetPhos were adjusted to reflect a ratio of 1:2 between the positive and negative set. This was performed by random removal of results from the positive or negative set, respectively. Subsequently, the AUCs were computed and compared. The obtained ROCs are provided in Additional file 1.

### Comparison to NetPhos, DisPhos 1.3 and KinasePhos 2.0 judged by accuracy, sensitivity, and specificity

As an alternative measure of performance, we also computed the accuracy defined as the proportion of correct predictions (true positive or true negative predictions) among the predictions made as well as the respective sensitivity, defined as the proportion of correctly classified positive sites and specificity, defined as the proportion of (Eq. 3):

(3)

where tp are true positive, tn-true negative, fp-false positive, and fn-false negative predictions.

The accuracy measure also allowed our prediction approach to be compared directly to other available prediction programs, especially KinasePhos 2.0. For computing accuracies, a decision threshold for the assignment of a site to a particular group must be set. The positive assignment threshold for our predictors was set to zero. Negative decision values were judged as predicted to be non-phosphorylated and positive decision values to be phosphorylated. For the other predictors, this value was set to 0.5 as they reflect probabilities. For kinase-specific predictions, sequences from training set were submitted to the KinasePho2.0 server. For assessing the performance associated with a particular kinase family, only the results corresponding to the particular family were evaluated as relevant predictions. As the prediction reports usually estimates the accuracies based on equal sizes of the positive and negative set, the negative sets were adjusted by random removal of the respective prediction results to reflect this ratio. This adjustment was performed 1,000 times with different random removals and the mean accuracy as well as the standard deviation was determined.

## Availability and requirements

The protein structure-based phosphorylation site prediction method developed as part of this study has been implemented as a freely accessible web-based service, Phos3D, and is available at .

## Abbreviations

AAindex: collection of 530 commonly used indices of amino acid properties; AUC: area under the ROC; PDB: protein data bank; PKA, PKB, PKG, PKC, RSK, CDK, CDC, CKII, ATM: kinase families; CMGC, AGC, CAMK: kinase groups; SVM: support vector machine; CV: cross-validation; FV: feature vector; ROC: receiver operating characteristic; RCP-Plot: Radial Cumulative Propensity Plot.

## Authors' contributions

PD, WW, JS, and DW conceived the study. PD and DW designed the analyses, interpreted the results, and wrote the manuscript. PD implemented all computational methods and carried out all computational analyses. CS developed and implemented the Phos3D server. JS helped revising the manuscript. All authors read and approved the final version of the manuscript.

## Supplementary Material

Additional file 1 **Additional Figures**. File contains a histogram of pairwise sequence comparisons of the protein sequences used in this study and ROC curves for Phos3D and other commonly used phosphorylation site prediction programs applied to serine, threonine, and tyrosine sites as well as selected kinase-specific sites. Click here for file

Additional file 2 **Training set for Phos3D**. File contains the positive and negative set from this study. Motifs as well as the associated PDB entries and annotated kinase family annotations are provided. Click here for file

## References

[B1] Denhardt DT (1996). Signal-transducing protein phosphorylation cascades mediated by Ras/Rho proteins in the mammalian cell: the potential for multiplex signalling. Biochem J.

[B2] Yaffe MB, Cantley LC (1999). Signal transduction. Grabbing phosphoproteins. Nature.

[B3] Nishida E, Gotoh Y (1993). The MAP kinase cascade is essential for diverse signal transduction pathways. Trends Biochem Sci.

[B4] Johnson LN, Barford D (1993). The effects of phosphorylation on the structure and function of proteins. Annu Rev Biophys Biomol Struct.

[B5] Obenauer JC, Cantley LC, Yaffe MB (2003). Scansite 2.0: Proteome-wide prediction of cell signaling interactions using short sequence motifs. Nucleic Acids Res.

[B6] Xue Y, Li A, Wang L, Feng H, Yao X (2006). PPSP: prediction of PK-specific phosphorylation site with Bayesian decision theory. BMC Bioinformatics.

[B7] Kim JH, Lee J, Oh B, Kimm K, Koh I (2004). Prediction of phosphorylation sites using SVMs. Bioinformatics.

[B8] Plewczynski D, Tkacz A, Godzik A, Rychlewski L (2005). A support vector machine approach to the identification of phosphorylation sites. Cell Mol Biol Lett.

[B9] Blom N, Sicheritz-Ponten T, Gupta R, Gammeltoft S, Brunak S (2004). Prediction of post-translational glycosylation and phosphorylation of proteins from the amino acid sequence. Proteomics.

[B10] Iakoucheva LM, Radivojac P, Brown CJ, O'Connor TR, Sikes JG, Obradovic Z, Dunker AK (2004). The importance of intrinsic disorder for protein phosphorylation. Nucleic Acids Res.

[B11] Diella F, Cameron S, Gemund C, Linding R, Via A, Kuster B, Sicheritz-Ponten T, Blom N, Gibson TJ (2004). Phospho.ELM: a database of experimentally verified phosphorylation sites in eukaryotic proteins. BMC Bioinformatics.

[B12] Jimenez JL, Hegemann B, Hutchins JR, Peters JM, Durbin R (2007). A systematic comparative and structural analysis of protein phosphorylation sites based on the mtcPTM database. Genome Biol.

[B13] Fan SC, Zhang XG (2005). Characterizing the microenvironment surrounding phosphorylated protein sites. Genomics Proteomics Bioinformatics.

[B14] Kemp BE, Pearson RB (1990). Protein kinase recognition sequence motifs. Trends Biochem Sci.

[B15] Pinna LA, Ruzzene M (1996). How do protein kinases recognize their substrates?. Biochimica et Biophysica Acta (BBA) – Molecular Cell Research.

[B16] Blom N, Gammeltoft S, Brunak S (1999). Sequence and structure-based prediction of eukaryotic protein phosphorylation sites. J Mol Biol.

[B17] Bagley SC, Altman RB (1995). Characterizing the microenvironment surrounding protein sites. Protein Sci.

[B18] Vapnik V (1995). The nature of statistical learning theory.

[B19] Joachims T, Schölkopf CB (1998). Making large-scale support vector machine learning practical. Advances in Kernel Methods: Support Vector Machines.

[B20] Wang Y, Tetko IV, Hall MA, Frank E, Facius A, Mayer KF, Mewes HW (2005). Gene selection from microarray data for cancer classification – a machine learning approach. Comput Biol Chem.

[B21] Qin J, Lewis DP, Noble WS (2003). Kernel hierarchical gene clustering from microarray expression data. Bioinformatics.

[B22] Pirooznia M, Deng Y (2006). SVM Classifier – a comprehensive java interface for support vector machine classification of microarray data. BMC Bioinformatics.

[B23] Vert JP (2002). Support vector machine prediction of signal peptide cleavage site using a new class of kernels for strings. Proc Symp Biocomput.

[B24] Zien A, Ratsch G, Mika S, Scholkopf B, Lengauer T, Muller KR (2000). Engineering support vector machine kernels that recognize translation initiation sites. Bioinformatics.

[B25] Burbidge R, Trotter M, Buxton B, Holden S (2001). Drug design by machine learning: support vector machines for pharmaceutical data analysis. Comput Chem.

[B26] Ding CH, Dubchak I (2001). Multi-class protein fold recognition using support vector machines and neural networks. Bioinformatics.

[B27] Heazlewood JL, Durek P, Hummel J, Selbig J, Weckwerth W, Walther D, Schulze WX (2008). PhosPhAt: a database of phosphorylation sites in Arabidopsis thaliana and a plant-specific phosphorylation site predictor. Nucleic Acids Res.

[B28] Schneider TD, Stephens RM (1990). Sequence logos: a new way to display consensus sequences. Nucleic Acids Res.

[B29] Hunter T (1987). A thousand and one protein kinases. Cell.

[B30] Manning G, Whyte DB, Martinez R, Hunter T, Sudarsanam S (2002). The protein kinase complement of the human genome. Science.

[B31] Kreegipuu A, Blom N, Brunak S (1999). PhosphoBase, a database of phosphorylation sites: release 2.0. Nucleic Acids Res.

[B32] Schwartz D, Gygi SP (2005). An iterative statistical approach to the identification of protein phosphorylation motifs from large-scale data sets. Nat Biotechnol.

[B33] Niefind K, Putter M, Guerra B, Issinger OG, Schomburg D (1999). GTP plus water mimic ATP in the active site of protein kinase CK2. Nat Struct Biol.

[B34] Reimer U, Reineke U, Schneider-Mergener J (2002). Peptide arrays: from macro to micro. Curr Opin Biotechnol.

[B35] Rychlewski L, Kschischo M, Dong L, Schutkowski M, Reimer U (2004). Target specificity analysis of the Abl kinase using peptide microarray data. J Mol Biol.

[B36] Mah AS, Elia AE, Devgan G, Ptacek J, Schutkowski M, Snyder M, Yaffe MB, Deshaies RJ (2005). Substrate specificity analysis of protein kinase complex Dbf2-Mob1 by peptide library and proteome array screening. BMC Biochem.

[B37] Linding R, Jensen LJ, Diella F, Bork P, Gibson TJ, Russell RB (2003). Protein disorder prediction: implications for structural proteomics. Structure.

[B38] Gnad F, Ren S, Cox J, Olsen JV, Macek B, Oroshi M, Mann M (2007). PHOSIDA (phosphorylation site database): management, structural and evolutionary investigation, and prediction of phosphosites. Genome Biol.

[B39] Zhou T, Sun L, Humphreys J, Goldsmith EJ (2006). Docking interactions induce exposure of activation loop in the MAP kinase ERK2. Structure.

[B40] Ptacek J, Devgan G, Michaud G, Zhu H, Zhu X, Fasolo J, Guo H, Jona G, Breitkreutz A, Sopko R (2005). Global analysis of protein phosphorylation in yeast. Nature.

[B41] Weckwerth W, Selbig J (2003). Scoring and identifying organism-specific functional patterns and putative phosphorylation sites in protein sequences using mutual information. Biochem Biophys Res Commun.

[B42] Cheng KY, Noble ME, Skamnaki V, Brown NR, Lowe ED, Kontogiannis L, Shen K, Cole PA, Siligardi G, Johnson LN (2006). The role of the phospho-CDK2/cyclin A recruitment site in substrate recognition. J Biol Chem.

[B43] Remenyi A, Good MC, Lim WA (2006). Docking interactions in protein kinase and phosphatase networks. Curr Opin Struct Biol.

[B44] Boeckmann B, Bairoch A, Apweiler R, Blatter MC, Estreicher A, Gasteiger E, Martin MJ, Michoud K, O'Donovan C, Phan I (2003). The SWISS-PROT protein knowledgebase and its supplement TrEMBL in 2003. Nucleic Acids Res.

[B45] Hanks SK, Quinn AM, Hunter T (1988). The protein kinase family: conserved features and deduced phylogeny of the catalytic domains. Science.

[B46] Thompson JD, Higgins DG, Gibson TJ (1994). CLUSTAL W: improving the sensitivity of progressive multiple sequence alignment through sequence weighting, position-specific gap penalties and weight matrix choice. Nucleic Acids Res.

[B47] Hooft RW, Sander C, Scharf M, Vriend G (1996). The PDBFINDER database: a summary of PDB, DSSP and HSSP information with added value. Comput Appl Biosci.

[B48] Sander C, Schneider R (1991). Database of homology-derived protein structures and the structural meaning of sequence alignment. Proteins.

[B49] Kabsch W, Sander C (1983). Dictionary of protein secondary structure: pattern recognition of hydrogen-bonded and geometrical features. Biopolymers.

[B50] PDBFINDER II database. ftp://ftp.cmbi.ru.nl/pub/molbio/data/pdbfinder2/.

[B51] Park Y, Helms V (2006). Assembly of transmembrane helices of simple polytopic membrane proteins from sequence conservation patterns. Proteins.

[B52] Levitt M (1978). Conformational preferences of amino acids in globular proteins. Biochemistry.

[B53] Alexandros K, Alexandros S, Kurt H, Achim Z (2004). kernlab – An S4 Package for Kernel Methods in R. Journal of Statistical Software.

[B54] Hanley JA, McNeil BJ (1983). A method of comparing the areas under receiver operating characteristic curves derived from the same cases. Radiology.

[B55] Kawashima S, Kanehisa M (2000). AAindex: amino acid index database. Nucleic Acids Res.

[B56] Wong YH, Lee TY, Liang HK, Huang CM, Wang TY, Yang YH, Chu CH, Huang HD, Ko MT, Hwang JK (2007). KinasePhos 2.0: a web server for identifying protein kinase-specific phosphorylation sites based on sequences and coupling patterns. Nucleic Acids Res.

[B57] DisPhos 1.3. http://core.ist.temple.edu/pred/pred/predict.

[B58] KinasePhos 2.0. http://kinasephos2.mbc.nctu.edu.tw/.

