# Supplementary material for: Detection and characterization of 3D-signature phosphorylation site motifs and their contribution towards improved phosphorylation site prediction in proteins
Source: BMC Bioinformatics. 2009 Apr 21;10:117. doi: 10.1186/1471-2105-10-117 (PMC2683816; doi:10.1186/1471-2105-10-117)

Supplementary Figure 1 Histogram of pairwise sequence comparisons of the 704 protein sequences used in this study. The dataset consisted of highly non-redundant protein sequences as evidenced by the low levels of sequence identities.

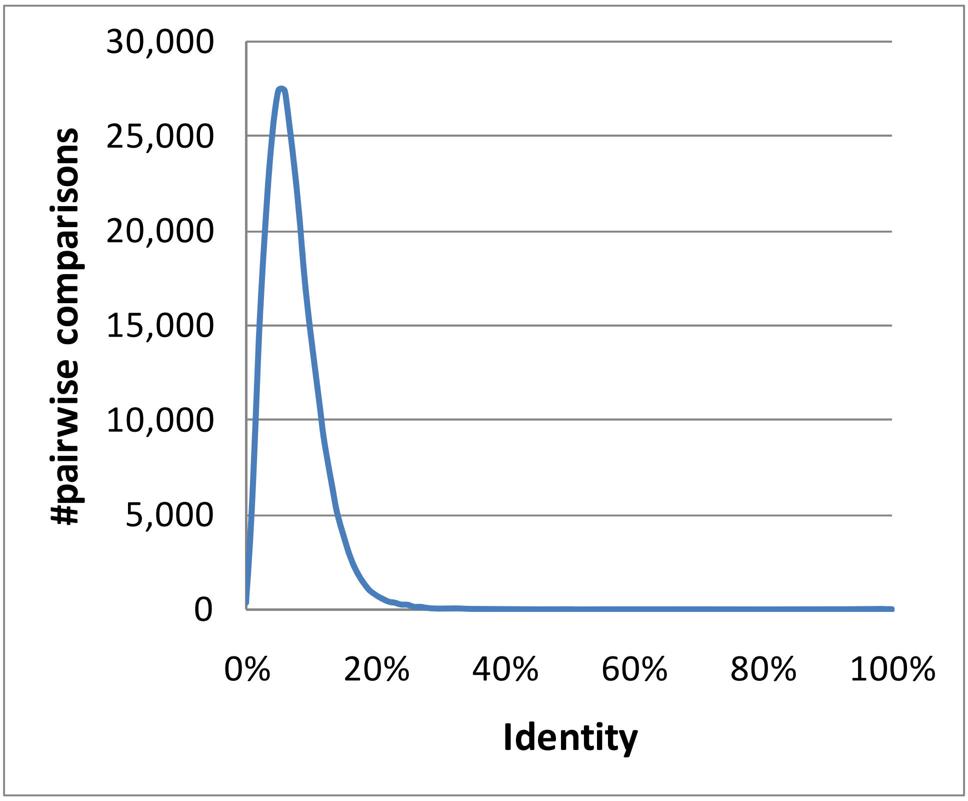

Supplementary Figure 2. Comparison of Receiver Operator Characteristics (ROCs) for Phos3D and other commonly used phosphorylation site prediction programs applied to serine, threonine, and tyrosine sites as well as selected kinase-specific sites as indicated in the respective legends.

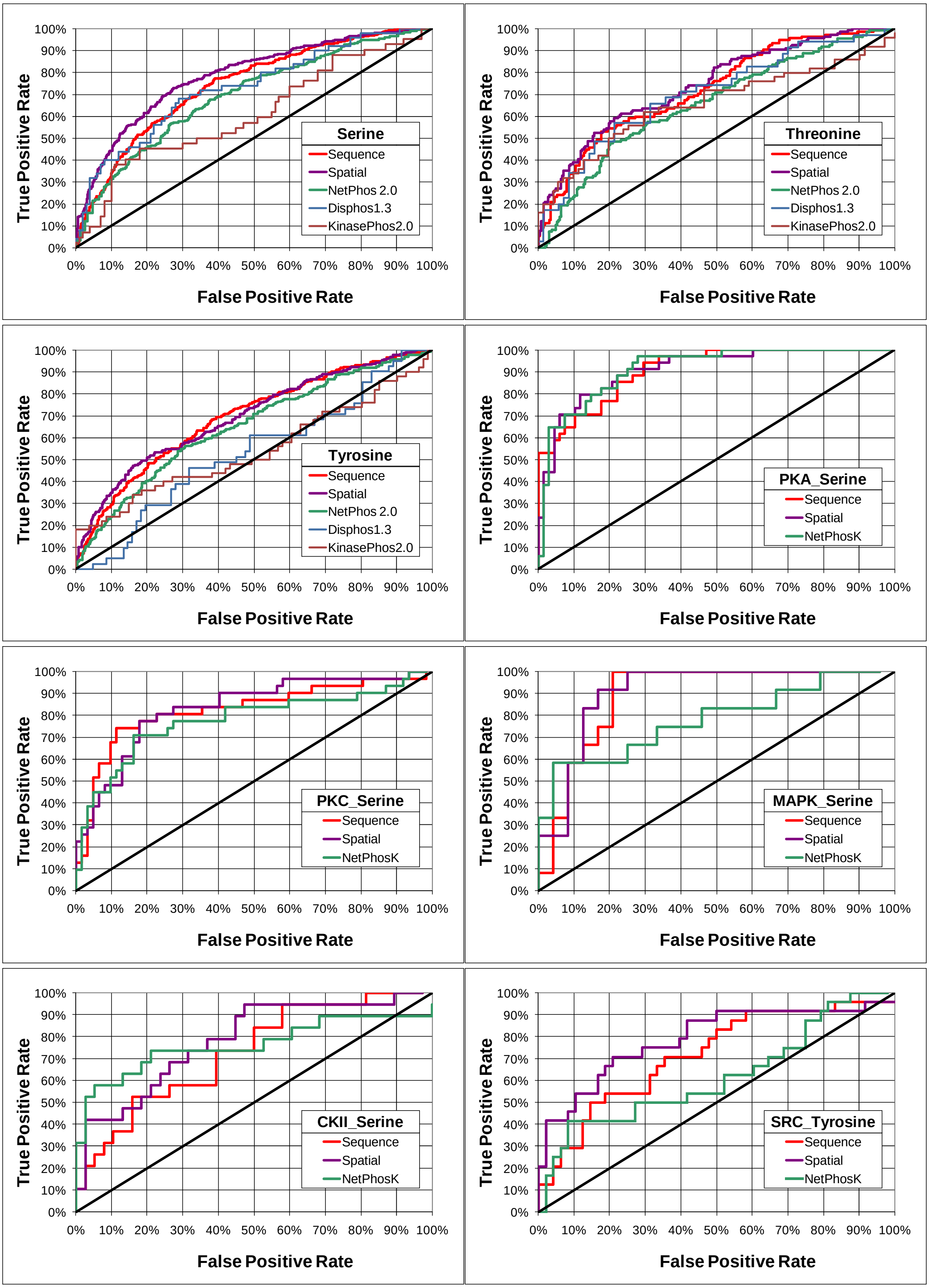

Supplement: Additional file 1 — Additional Figures. File contains a histogram of pairwise sequence comparisons of the protein sequences used in this study and ROC curves for Phos3D and other commonly used phosphorylation site prediction programs applied to serine, threonine, and tyrosine sites as well as selected kinase-specific sites. [file 1471-2105-10-117-S1.pdf]
